# A comprehensive review of goji berry processing and utilization

**DOI:** 10.1002/fsn3.3677

**Published:** 2023-10-13

**Authors:** Jing Yu, Yamei Yan, Lutao Zhang, Jia Mi, Limei Yu, Fengfeng Zhang, Lu Lu, Qing Luo, Xiaoying Li, Xuan Zhou, Youlong Cao

**Affiliations:** ^1^ College of Light Industry and Food Science Zhongkai University of Agriculture and Engineering Guangzhou China; ^2^ Institute of Wolfberry Engineering and Technology Ningxia Academy of Agriculture and Forestry Yinchuan China; ^3^ Ningxia Agricultural Products Quality Standards and Testing Technology Research Institute Yinchuan China

**Keywords:** by‐products, comprehensive utilization, efficacy components, goji berry, processing

## Abstract

Goji berry (wolfberry, *Lycium*), is a genus of Solanaceae, in which the roots, stems, leaves, and fruits are for both food and medicinal uses. In recent years, the demand for health food and research purposes has led to increasing attention being paid to the application of goji berry nutrients and resources. There are three general strategies to process and utilize the goji berry plant. First, the primary processing of goji berry products, such as dried goji berry pulp, and fruit wine with its by‐products. Second, deep processing of sugar–peptides, carotenoids, and the extraction of other efficacy components with their by‐products. Third, the utilization of plant‐based by‐products (roots, stems, leaves, flowers, and fruit residuals). However, the comprehensive use of goji berry is hampered by the non‐standardized production technology of resource utilization and the absence of a multi‐level co‐production and processing technology systems. On the basis of this, we review some novel techniques that are made to more effectively use the resources found in goji berry or its by‐products in order to serve as a guide for the thorough use of these resources and the high‐quality growth of the goji berry processing industry.

## Graphical abstract

1. The utilization and development of Goji berry including the roots, stems, leaves, and fruits, all of which are for both food and medicinal uses.
2. Three general strategies to process and utilize the goji berry plant: the primary processing products, deep processing, and the plant‐based goji berry by‐products and their utilization.
3. Some novel techniques that are made to more effectively use the resources found in goji berry, such as tissue culture and synthesis in the application of the active ingredients

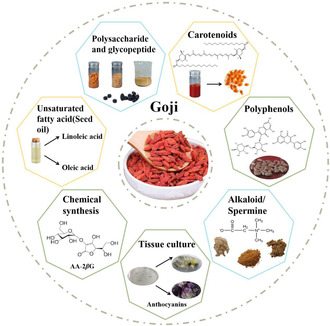

## 1 INTRODUCTION

**FIGURE 1:**
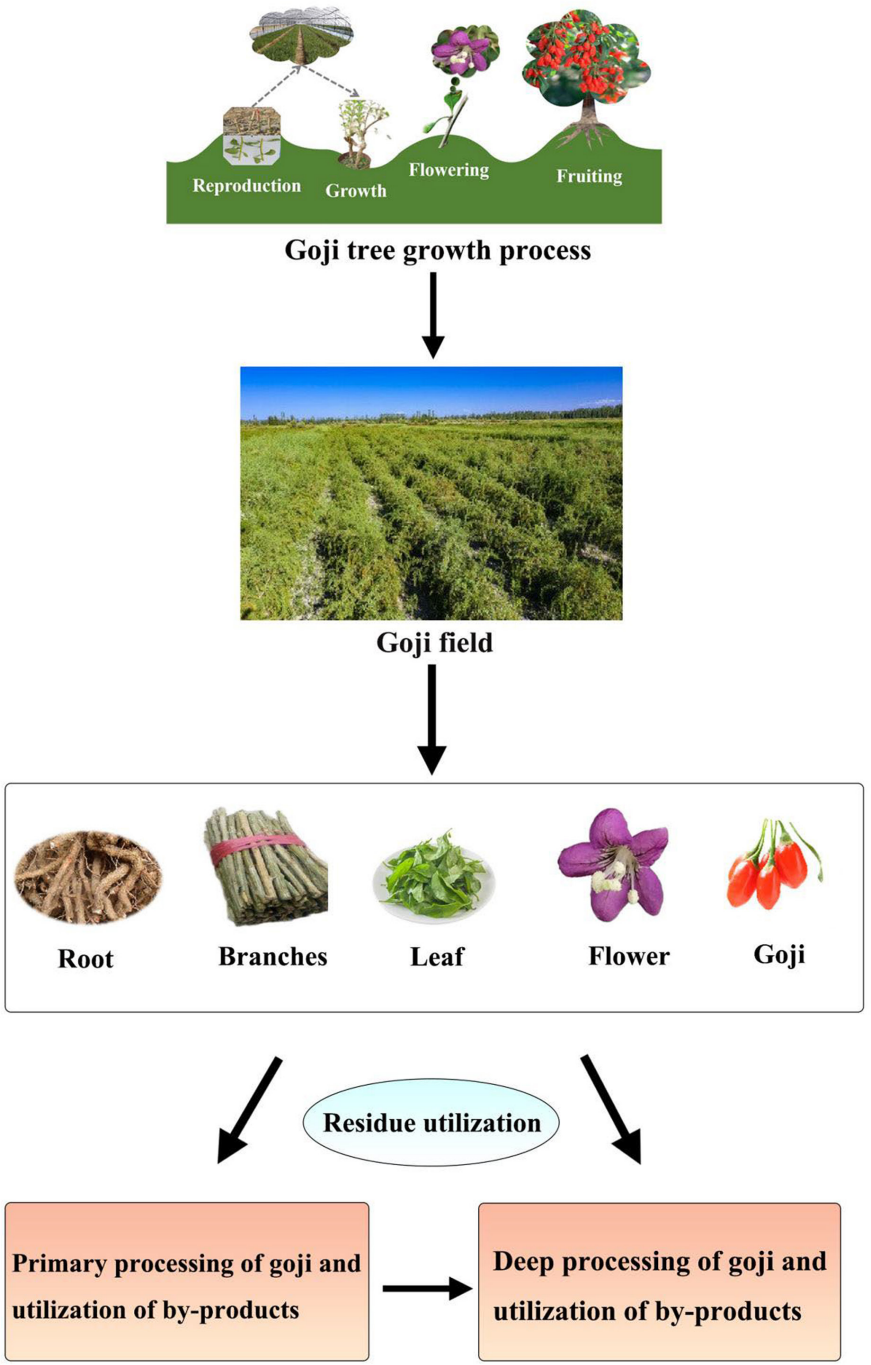
Overview of the comprehensive utilization of Goji berry.

Goji berry (wolfberry, *Lycium*), is a genus of Solanaceae. The term “*goji*” in Chinese language includes a variety of species including *Lycium barbarum* L., *Lycium chinense* Mill., and *Lycium ruthenicum* Murr. This genus of ancient origin is widely distributed in Asia, Africa, America, and Europe. In addition to its economic and medicinal values, goji berry can be also used for windbreaking and saline soil conservation because of its high salt tolerance and biological drainage abilities. It has a cultivation history of more than 2300 years mainly in Ningxia and other provincial units in Northwest China (Ciceoi et al., 2021; Skenderidis et al., 2018; Zhao et al., 2016). The most commonly grown goji berry variety in China is *Lycium barbarum* L., one of the currently recognized species. Its fruit has been found to contain a range of health‐promoting ingredients, including carotenoids, polysaccharides, polyphenols, amides, 2‐*O*‐β‐d‐glucopyranosyl‐l‐ascorbic acid (AA‐2βG), etc., presenting the effects of eye health protection, blood lipid reduction, antioxidation, and anti‐aging (Skenderidis et al., 2018). Apart from the fruit, the roots, stems, leaves, pollen and other by‐products of goji berry can also be used as outstanding edible or medicinal resources. Figure 1 presents the current three types of goji berry utilization. The first type involves primary processing of goji berry and its by‐products, such as fruit juice production, fruit wine, and other beverages, and processing of seed skin residue and other by‐products. The second type involves deep processing of goji berry, including the isolation of glycopeptides, carotenoids, and other potent ingredients and their by‐products. The third type involves the collection of goji berry roots, stems, leaves, and bee pollen during the growing process. In recent years, with the awareness of health benefits of goji berry, the goji berry planting area and production output have significantly increased. As of 2022, the harvesting area goji berry reached 380,000 *mu* (62,600 acres), and the total fresh fruit output was 300,000 tons with its processing conversion rate being 30% solely in Ningxia, a northwestern Chinese autonomous region (Mao, 2022). Ningxia's goji berry industry has the core production area with the best foundation, the most complete production factors, the strongest scientific and technological support, and the most prominent brand advantage (Ren & Bai, 2022; Wang, 2022). With the booming goji berry industry, methods to comprehensively and efficiently use the goji berry resources have become very important to the development of goji berry processing industry in China.

This work presents a review on the nutrition, efficacy, resource utilization, and development trends of the goji berry industry. It covers primary processing of goji berry, such as raw goji berry pulp and fruit wine, as well as deep processing of goji berry fruit and its potent ingredients, including glycopeptides and carotenoids, and reviews the utilization of plant‐based goji berry products.

## 2 PRIMARY PROCESSING OF GOJI BERRY FRUIT AND ITS BY‐PRODUCTS

### 2.1 Primary processed products

**FIGURE 2:**
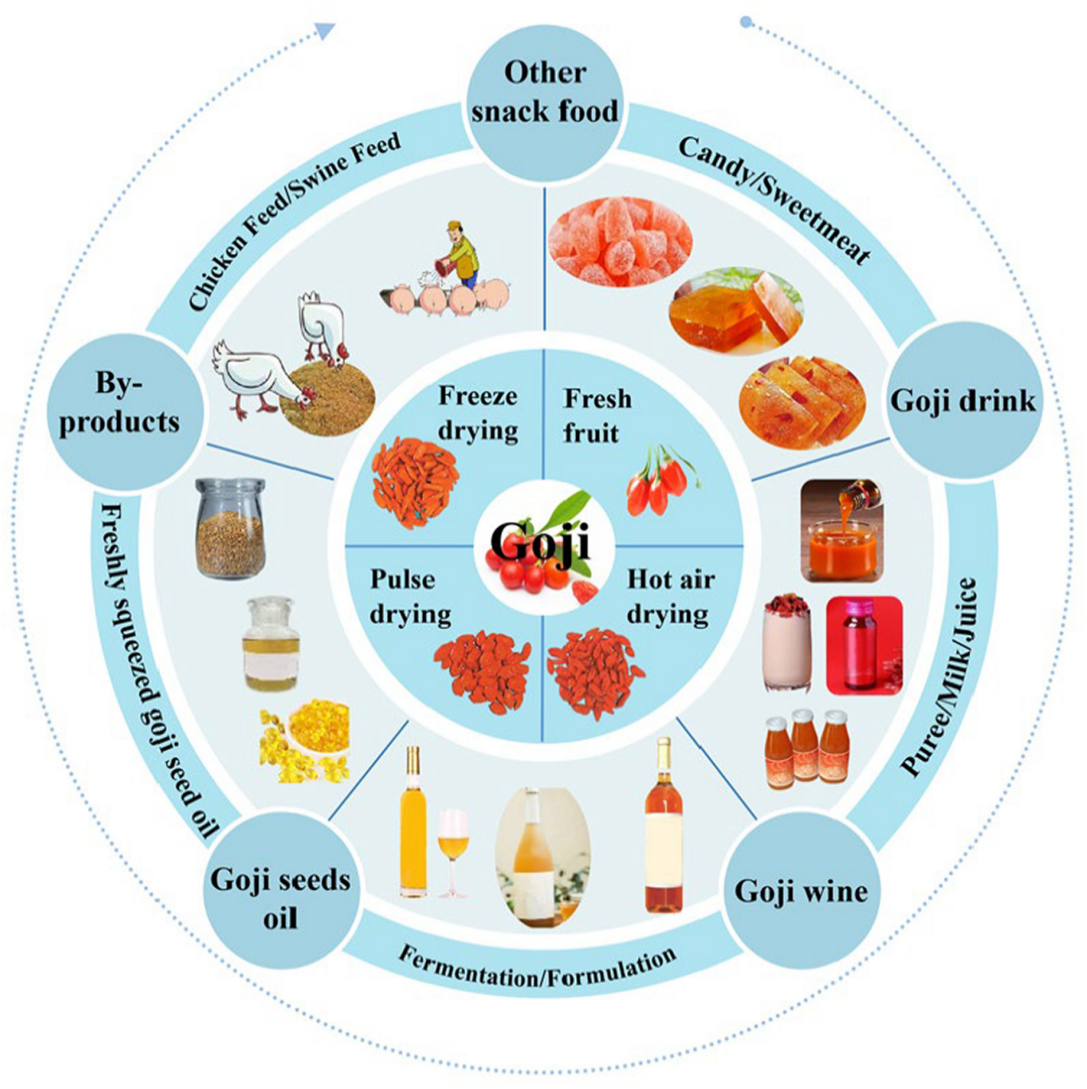
Primary processing and the by‐product utilization of Goji berry.

The primary processing of goji berry after the harvest is shown in Figure 2. About 75%–85% of fresh goji berry fruit produced are dehydrated by traditional hot air drying, lyophilization, or vacuum pulsation drying technologies before being put on the market (Batu & Kadakal, 2021; Ni et al., 2020; Yu et al., 2020). Apart from the dried fruit, goji berry juice, wine, and other primary products have become the main varieties of goji berry products. Goji berry juice includes pulp juice, clear juice, solid beverages, milk beverages, lactic acid fermented beverages, etc. (Braga et al., 2019; Liu, Meng, et al., 2020; Liu, Cheng, et al., 2020; Wang, Ouyang, et al., 2021). Goji berry wines include blended wines that are made by infusing goji berries and other active ingredients or medicinal herbs into distilled spirits, and fermented goji berry wines that are mainly made of fermentation of goji berries along with *Ziziphus jujuba* Mill. (dates), honey, and other nutritious food (Geng et al., 2021). Among them, goji berry pulp is a rapidly rising variety of processed products in recent years, which is made from fresh goji berry fruit as the only raw material through beating, rough filtration, homogenization, pasteurization, filling, and other processes so as to maintain the nutritional composition and flavor of fresh goji berry fruit to the greatest extent. By 2020, there are 10 goji berry pulp production lines, 32 packaging lines, and the production capacity of goji berry pulp reaches more than 10,000 tons. At the same time, the national and local governments of China have issued an industrial standard GH/T 1237–2019 and an association standard T/NXFSA 002S‐2020 of goji berry pulp (Zhang et al., 2022). According to the statistics, the annual sales of goji berry pulp reached 1 billion RMB (Geng et al., 2021). Also, goji berry powder, goji berry honey, goji berry oatmeal, goji berry coffee, goji berry chocolate, and other snacks have entered the market (Yu, 2023).

### 2.2 Primary processing by‐products

The by‐product utilization of goji berry after the harvest is showed in Figure 2. The production processes of goji berry juice and goji berry wine produce a large amount of pomace including flesh, peel, and seeds, the comprehensive utilization of by‐products is as follows: First, the by‐products can be used as feeds and organic fertilizers. Given the juice yield of goji berry is about 50%–80% w/w, a large amount of peel residue can be produced after primary processing. Wang et al. conducted experimental research and found that incorporating 5% goji berry residue into a feed pellet mixture improved nutrient digestibility in the diet of fattening goats, while also enhancing their antioxidant capacity and immune performance (Wang, Ouyang, et al., 2021; Wang, Qi, et al., 2021). Similarly, Abdallah et al., (2019) noted that goji berry residue is a green feed ingredient that could potentially replace antibiotics in animal husbandry to improve animal performance. The by‐products can also be developed as organic fertilizers. Zhang et al. have developed a method for producing organic fertilizer by fermenting the by‐products of polysaccharide extraction from *Lycium barbarum* L. and fennel oil extraction, along with liquid traditional Chinese medicine and other ingredients (Zhang, 2018). Second, it can be used to extract the goji berry seed oil. The mixture of goji berry peel and goji berry seeds is obtained after pressing the fresh fruit of goji berry. Goji berry seeds make up approximately 3% w/w of the fruit, and contain about 25% w/w of oil, with unsaturated fatty acids accounting for up to 90% w/w of the oil content. Thus, these seeds are excellent oil resources (Ma, 2014; Pedro et al., 2019). Li et al. have developed a separation technique that is more effective in removing goji berry seeds from goji berry fruit residue, while also preserving the quality of products made from goji berry fruit juice (Li, 2011). The recovery rate can be as high as 97%. The extraction rate is 94% when using supercritical CO_2_ to extract *Lycium barbarum* L. seed oil. Therefore, one of the significant current uses of *Lycium barbarum* L. peel residue by‐products is *Lycium barbarum* L. seed oil refined by extraction or pressing. Third, for the development and utilization of dietary fiber and other components (Gong, 2020). Sun et al., (2013) showed that dietary fiber can be isolated from the residue after the extraction of polysaccharides from the fruit of goji berry. Bora et al., (2019) developed muffins and cookies with significantly improved textures and organoleptic properties by replacing wheat flour with goji berry pomace. Goji berry pomace is rich in pectin polysaccharides, phenolic substances, carotenoids, etc. from which a large number of active extracts are further utilized Amides have been observed in goji berry (El Kantar et al., 2020; Liu, 2015; Men et al., 2021; Wang et al., 2014). However, there is little information of the amides in the pomace of goji berry (Qian et al., 2020).

## 3 DEEP PROCESSING OF GOJI BERRY FRUIT AND ITS BY‐PRODUCTS

**FIGURE 3:**
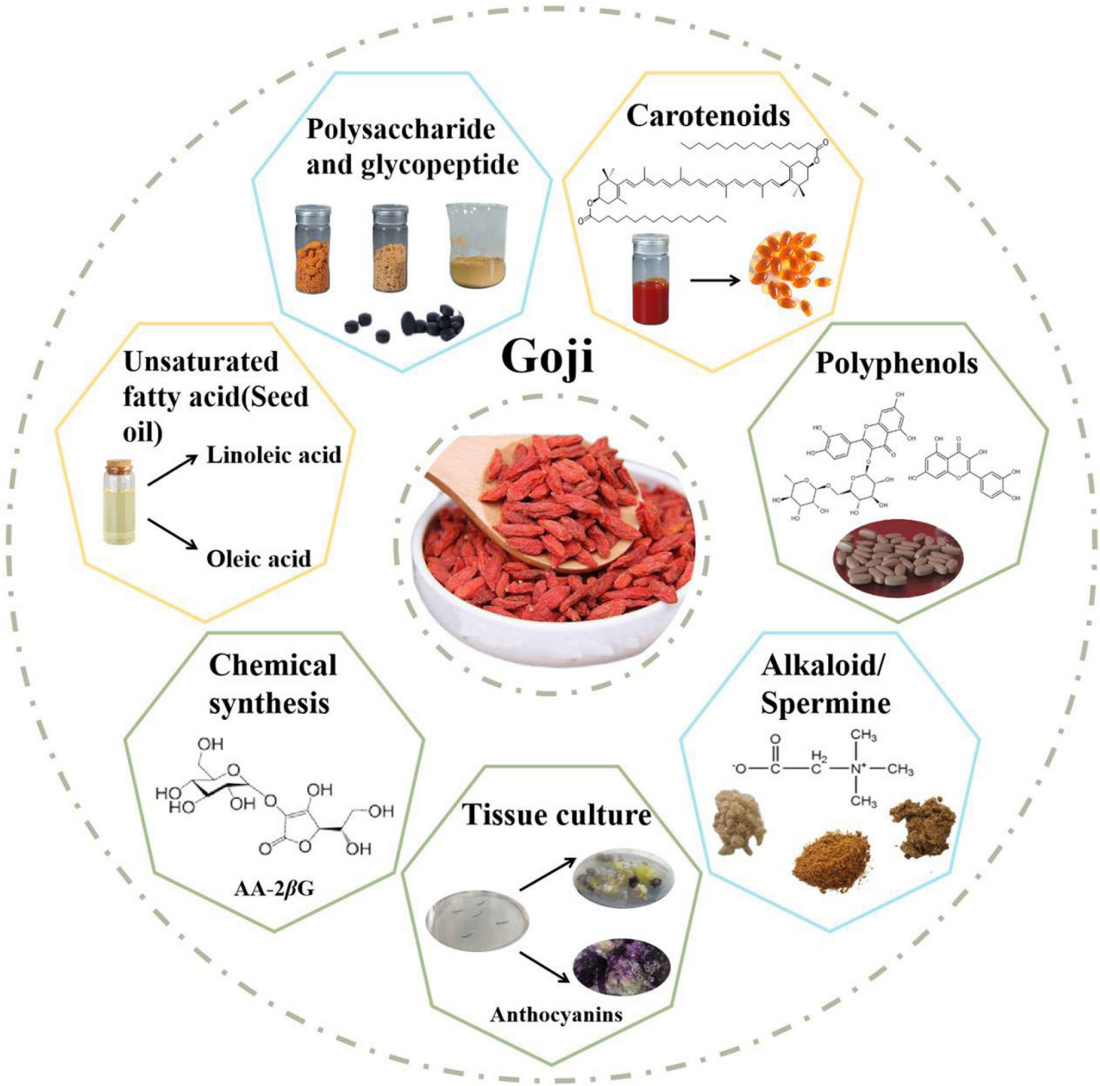
Deep processing and the utilization of the by‐products of Goji berry.

The deep processing utilization of goji berry is shown in Figure 3, the extraction, synthesis, and utilization of some functional ingredients, such as polysaccharides/glycopeptides, carotenoids, polyphenols, alkaloids, and unsaturated fatty acids in goji berry are exploited.

### 3.1 Extraction and utilization of the polysaccharides/glycopeptides in goji berry

*Lycium barbarum* L. polysaccharides (LBPs) are among the most effective components of goji berry, with high‐scale production and utilization. These products have been recommended by hospitals as a source of nutrients (Tian et al., 2019; Yang et al., 2015). LBPs are complex mixture of highly branched and only partially characterized polysaccharides and proteoglycans with molecular masses of 10–2300 kDa. Neutral sugars, such as xylose, mannose, arabinose, rhamnose, galactose, and glucose, are among the main components of LBPs. A neutral polysaccharide known as LBP‐1 has exhibited remarkable anti‐tumor activity by inhibiting the growth of A549 tumor cells, blocking the cellular G0/G1 phase, and regulating the PI3K/Akt/mTOR signaling pathway to induce apoptosis. LBP‐1 is composed of arabinose, galactose, glucose, xylose, and mannose. The ratio of the amount of substances was 37.53*:* 28.08: 14.72: 7.83: 4.50. (Ma et al., 2022) Another neutral polysaccharide, LBP3b, was isolated by Tang et al. using ultrafiltration membrane isolation, which exhibited hypoglycemic effects by inhibiting glucose uptake. LBP3b is composed of mannose, rhamnose, glucose, galactose, and xylose, with a substance ratio of 5.52: 5.11: 28.06: 1.00: 1.70, covalently linked with a small amount of protein (Tang et al., 2015; Yang et al., 2015).

There are a large number of studies on the application of single extraction or compound extraction by leaching, enzymatic, microwave, subcritical extraction, and ultrasonic methods (Tian et al., 2019; Yang et al., 2015). However, the extraction studies mainly focus on the extraction yields of LBPs. Yang et al. compared the extraction yields and activity differences between hot water (100°C), ultrasonic water (30–40°C), subcritical water (110°C), and ultrasonic enhanced subcritical water extraction (110°C). The results showed that the total yields of LBPs ranged from 7.6% w/w to 14% w/w (Yang et al., 2015). The protein contents, total phenolic contents, and in vitro activities of the polysaccharides extracted by different methods also differ significantly. Zhou et al. (2020) used water extraction at different temperatures assisted by acid or base to extract LBPs, which provided further improved purifications. LBPs obtained by high‐temperature acidic extraction contained more homogalacturonic acid regions and lost most of its side chains, especially arabinan. On the other hand, the polysaccharides extracted by base produced a large‐size polymer in branched form through SEC‐MALLS and AFM analysis. Yao et al. (2018) discovered that the antioxidant activity of *Lycium barbarum* pectin polysaccharide PLBP‐II‐I was more pronounced than PLBP‐I‐I analysis of the monosaccharide composition revealed that the content of galacturonic acid was higher in PLBP‐II‐I, and this difference in structure resulted in a significant difference of activity. The complexity of nonhomogeneous structures of LBPs, such as monosaccharide compositions, chain conformations, ring conformations, and various molecular weights, and their activities are greatly influenced by the extraction methods (Masci et al., 2018).

However, there is no uniform standard for the quality control of the LBPs production process. The total contents of polysaccharide and protein of goji berry are regulated to ≥25.0 g/100 g in Social Organization Standard of China T/CCCMHPIE 1.77—2022 (China Chamber of Commerce for Import & Export of Medicines & Health Products, 2022). There is a great progress in protein characterization compared with the determination of LBPs in the 2020 Chinese Pharmacopeia, but the characteristic indexes related to the structure of polysaccharide and glycopeptide have not been included in the standard, providing less specificity in identification of LBPs. Furthermore, the evaluation criteria for LBPs solely relies on the measurement of glucose levels (Chinese Pharmacopoeia Commission, 2020), the quantitative analysis of the total polysaccharide makes the quality control far from the industrialization demand of LBPs.

### 3.2 Extraction and utilization of carotenoids from goji berry

Carotenoids are an important group of natural pigments that make up the primary component of fruit and vegetable colors. These compounds are classified as C40 terpenoids and their derivatives, consisting of ‐ isoprene units linked head‐to‐tail, with the central region of the molecular formula containing 9–13 conjugated double bonds formed by the isoprene chain (Bonet et al., 2020; Britton, 2020). Due to their multiple conjugated double bond structure, carotenoids exhibit a range of colors. For example, β‐cryptoxanthin appears orange, zeaxanthin appears orange‐yellow, and β‐carotene appears orange. There is a correlation between the content and composition of carotenoids and the color of appearance.

Carotenoids are among the primary coloring substances and active components in goji berries. Studies have demonstrated that mature fruits of *Lycium barbarum* L. accumulates a significant amount of carotenoids, with content ranging from 120 to 400 mg/100 g. The carotenoid contents of ripe goji berry fruit range from 120 to 400 mg/100 g (Mi et al., 2018; Ordoñez‐Quintana et al., 2020). The main compound in goji berry is zeaxanthin dipalmitate (ZDP), which is also known as “goji berry red pigment” according to So et al. (Xiao et al., 2017). ZDP has become a recent topic of interest due to its various efficacies, including antioxidant properties and protection of the eyes, as well as prevention of liver damage induced by chronic hepatitis B and non‐alcoholic fatty liver disease (Bahaji Azami & Sun, 2019; Liu et al., 2021; Yan et al., 2018). Given that goji carotenoids are mainly fat‐soluble and have low polarity, the extraction and preparation processes have developed in recent years from solvent methods to supercritical and subcritical extraction techniques. Due to the large amount of fructose and glucose, or other small molecule sugar, the extracted residues stick together when the extraction temperature reaches 35–45°C and stay adamantly on the surface of the extraction vessel. These residues cannot be removed, resulting in damages to the extraction vessel. The larger the scale of production, the more serious the phenomenon. Our group used a sugar reduction process to mitigate this impact (Mi et al., 2021). Firstly, soaking the dry goji berries with water at a lower temperature, reducing the total sugar contents from 41%–45% to 25%–29%. Secondly, the desugarized wolfberries are dried again to reduce the raw material's water content from 11%–12% to 8%–10%. Finally, the dried goji berries were crushed and placed in a supercritical extraction vessel, and the purity of the zeaxanthin dipalmitate extract could reach more than 80% (Yan et al., 2017). The technical parameters are suitable for pilot scale up productions. As one of the main efficacy components of goji berry, carotenoids have potential physiological functions and health care values. In general, however, there is limited research and development application in the deep processing of goji berry carotenoids. The in vivo metabolic pathways of zeaxanthin dipalmitate in goji berry need to be further explored.

### 3.3 Extraction of polyphenols and other active ingredients of goji berry

Polyphenolic compounds are one of the main active components of goji berry and have good biological and pharmacological activities (Rocchetti et al., 2018). Macroporous resins and membrane separation are used for the separation of smaller amounts of polyphenolic compounds in goji berry. Liu et al. developed mixed‐mode macroporous adsorbent resins (MARs) and increased the total flavonoid content of purified flavonoid (p‐FLA) extracts from 0.97% to 36.88% (Liu, Cheng, et al., 2020; Liu, Meng, et al., 2020). Conidi et al., (2022) evaluated the potential of three compact ultrafiltration (UF) membranes with molecular weight cutoffs (MWCOs) ranging from 1.0 to 3.5 kDa for the separation of phenolic compounds from the aqueous extraction of glycoconjugates. The 2.5 kDa membrane allowed to reduce the total carbohydrate concentration from 26.5 g to 2.2 g glucose/L (91.7% reduction) during the percolation/batch concentration process and to obtain a concentrated extract rich in phenolic compounds.

Beside the above polysaccharides, carotenoids, and polyphenolic compounds, spermine alkaloids are important bioactive components in goji berry that have been newly discovered (Table 1). Ahad et al. conducted a chemical analysis of spermine in goji berry by UPLC‐Q‐TOF/MS. Four structural types of standards were used to study the integrated cleavage pattern of spermine (Ahad et al., 2020). The different types of spermine were identified by unique MS/MS fragment ions. For the first time, the coexistence of fragment ions at m/z 220 and 222 were proposed as a key feature to distinguish the sub‐spermine isomers. Based on the structural characteristics of spermine, a rapid, convenient, and highly selective strong cation exchange solid phase extraction (SCX‐SPE) combined with RP‐LC was developed for the selective enrichment and MS detection compatibility of spermine. However, the low content and the lack of UV absorption of spermidine make it difficult to quantify. As a result, it has yet been applied. Also, Ji et al. isolated three novel LBP cyclic peptides (GCP) and determined the amino acid sequences. Among them, GCP‐1 (Cycle‐(Trp‐Glu‐His‐Thr)) inhibited proliferation and induced apoptosis in human cervical cancer (HeLa) cells and significantly blocked G0/G1 phase HeLa cells (Ji et al., 2021). GCP‐1 also inhibited the growth of cervical cancer in vivo. Bubloz et al. (2020) evaluated and confirmed the presence of AA‐2βG in the fruit, rhizomes, stems, and leaves of goji berry. However, it was 40–280 mg/100 g dry weight in fruit, much higher than in other tissues. In short, these active ingredients such as spermine, cyclic peptides, and ascorbic acids are presented as trace amounts in goji berry, but whether they can be enriched in the above polysaccharide or polyphenol/flavonoid extracts and exert active effects based on the principle of polarity or similarity of properties needs to be further explored.

**TABLE 1:** Summarizes various extraction methods of goji berry active ingredients.

Types of ingredients	Extraction methods	Solvent	Contents (mg/g) or yields (%)	References
LBPs	Heating	Water	7.46%–7.63%	Li et al. (2007); Luo et al. (2000); Yang et al. (2015)
	Subcritical medium	Water	10.67 ± 0.33%	Yang et al. (2015)
	Ultrasound‐assisted	Water	2.286%–5.701%	Muatasim et al. (2018); Skenderidis et al. (2018)
	Enzyme‐assisted	Water	6.81 ± 0.10%	Liu et al. (2013)
	Ultrasound‐enhanced subcritical	Water	2.286%‐5.957%	Zhao et al. (2013)
Fat‐soluble substances	Supercritical medium	CO_2_	8.55%	Mi et al. (2021)
Total carotenoids			37.64 mg/g	
ZDP			29.54 mg/g	
Total flavonoids	Mix‐mode macroporous adsorption resins (MAR)	–	36.88% of p‐FLA	Liu, Meng, et al., (2020)
	High‐speed shear dispersing emulsifier	Deep eutectic solvents	7.11 mg/g	Wang et al., 2022)
Spermine alkaloids	Ultrasound assisted	Methanol	16.67% of 5 g sample	Ahad et al. (2020)
GCP	Solvation and ultra‐filtration	PBS (0.5 M)	–	Ji et al. (2021)
AA‐2βG	Ultrasound‐assisted	Oxalic acid/water	22% in rhizomes, 14% in stems, 14% in leaves (dry weight)	Bubloz et al. (2020)

### 3.4 Application of tissue culture and chemical synthesis for producing active ingredients in goji berry

Micropropagation of *Lycium barbarum* L. is both time consuming and labor‐intensive, as well as expensive. However, Ruta et al. were able to achieve large‐scale production of high‐quality goji berry buds using the bioreactor plant morphology TM. Furthermore, they assessed this method to produce total phenols and flavonoids (Ruta et al., 2020). The outcomes demonstrated that the three soaking cycles had various impacts on the development and hydration of buds. This culture produced higher levels of total phenols and lower levels of total flavonoids as compared to semi‐solid culture. Karakas, (2020) developed the in vitro culture protocols for healing tissue induction and plant regeneration from different explants of goji plants and compared the phenolic composition of healing tissues from different sources. The results revealed that the phenolic compositions and contents of the healing tissues obtained with different plant growth regulators (PGR) or combinations differed. The combination of BA/NAA significantly increased the production and accumulation of chlorogenic and caffeic acids. The combination of TDZ/IAA, TDZ alone, and TDZ/NAA significantly increased the synthesis of vanillic and rutin, gallic acid and quercetin, respectively. These results suggest that different PGR result in the production of different kinds of secondary metabolites and affect/accelerate the accumulation in the healing tissues of goji.

**SCHEME 1:**
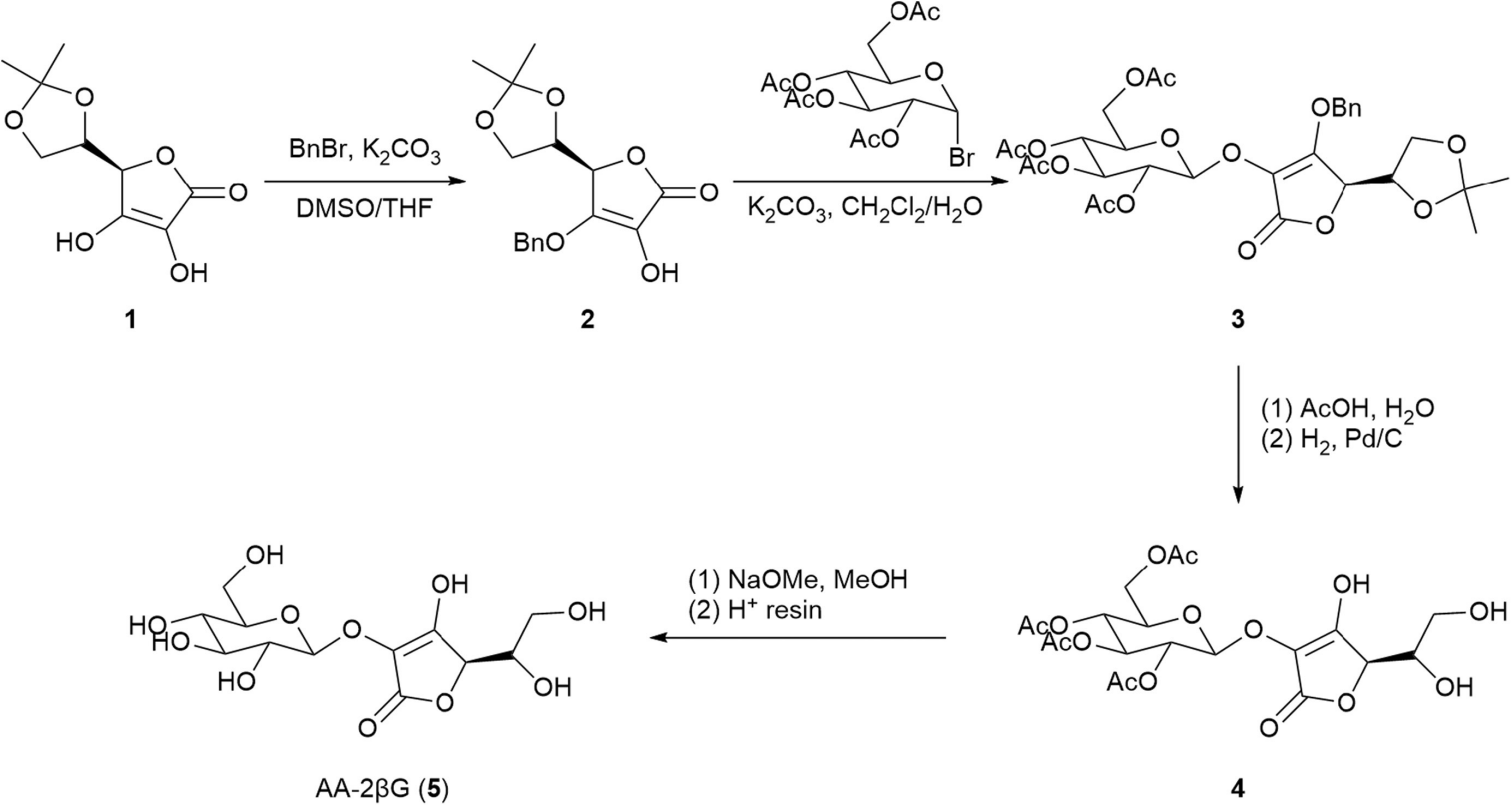
Synthetic pathway of AA‐2βG.

The artificial synthesis of goji berry natural compounds have been widely explored over the decades. Methods on the chemical synthesis of the active ingredient 2‐*O*‐β‐d‐glucopyranosyl‐l‐ascorbic acid (AA‐2βG) in goji berry fruit have been well developed. AA‐2βG was initially isolated from Ningxia wolfberries and lab‐synthesized by Japanese researchers Toyoda‐Ono et al. (2004). In 2017, Ma et al. (2017) upgraded the preparation method of AA‐2βG. The synthetic pathway of AA‐2βG is shown in Scheme 1. First, commercially available 5,6‐*O*‐isopropylidene‐l‐ascorbic acid (**1**) with propylene protection is benzylated at the most active 3‐hydroxyl position to give the compound **2**. Then, the glycosylation reaction of **2** is conducted at the 2‐hydroxyl position by glucosyl bromide in a CH_2_Cl_2_/water solution, giving the compound **3**. The benzyl and the propylene protection groups in **3** are subsequently removed, which gives **4**. Finally, AA‐2βG (**5**) is obtained via deacetylation on the glucosyl ring in **4**. AA‐2βG can only be digested by ‐glycosidase in the small intestine and releases ascorbic acid slowly, which can accomplish continuous free radical scavenging and antioxidant effects and can also stop Hela cells from proliferating and growing (Takebayashi et al., 2008; Zhang, Liu, Wu, et al., 2011; Zhang, Liu, Zhang, et al., 2011). Huang et al.'s (2019) results of their study showed that AA‐2βG can treat inflammatory bowel disease (IBD) caused by DSS, as well as reduce body weight, enhance serum physiological and biochemical indicators, lengthen the colon, promote the production of short‐chain fatty acids, and regulate the composition of intestinal flora.

Peng et al. (2021) explored the possibility of biosynthesizing LBPs through yeast, but the synthetic completion pathway and key enzymes of LBPs are unknown, limiting the production of LBPs by yeast. Finding the key enzymes and designing the complete pathway using histological data is a possible way to achieve large‐scale production of LBPs.

As mentioned above, the LBP and small molecules such as carotenoids, phenols, and amides that can be extracted and exploited in goji berry currently vary widely in molecular weight and polarity, as well as in extraction techniques and activities. Traditional Chinese medicine is characterized by multiple active ingredients, multiple links, and multiple targets (Fan et al., 2021). However, no studies on the overall effects of various ingredients of goji berry have been reported. There is no research on the co‐extraction technology of various efficacy components, and the extraction of single components causes waste of resources and low added value, which further affects deep processing of goji berry. It is lack of the application of a variety of technologies integrated process especially, such as membrane separation and resin adsorption, the combination of supercritical fluid extraction and chromatography, adsorption clarification‐high‐speed centrifugation‐membrane separation process.

## 4 PLANT‐BASED GOJI BERRY BY‐PRODUCTS AND THEIR UTILIZATION

**FIGURE 4:**
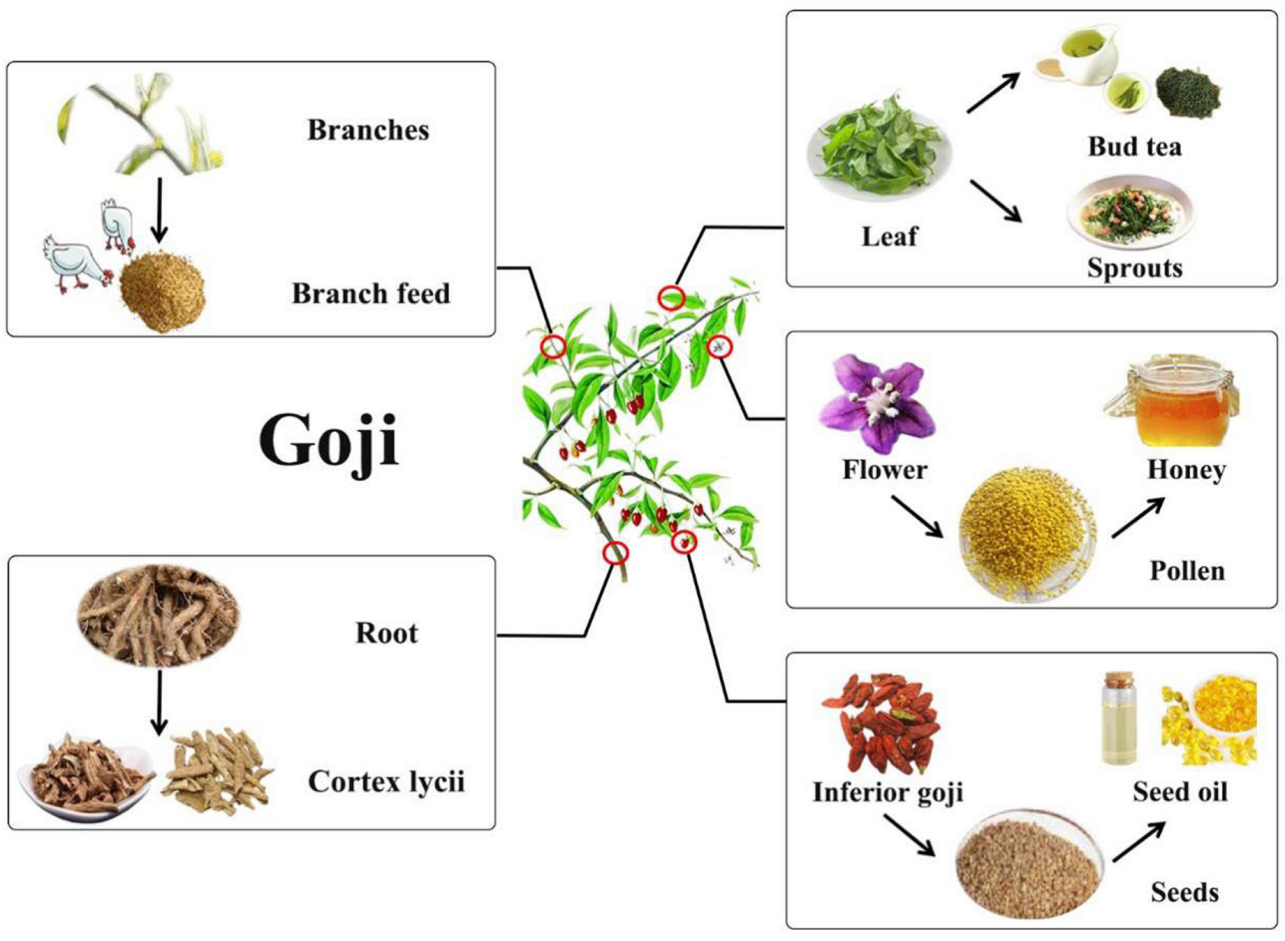
Planting by‐products and their utilization of Goji berry.

Plant‐based goji berry by‐products, for example, roots, stems, leaves, and fruit residuals, have been widely applied (Figure 4) throughout the decades.

### 4.1 Goji berry roots

*Cortex Lycii Radicis* (CLR), the roots of goji berry have a long history of use. Chinese Pharmacopeia records the dried root bark of *Lycium barbarum* L. or *Lycium chinense* Mill. as one kind of Chinese herb (Qian, 2016). After being harvested in early spring or after autumn, the roots are washed, stripped of the root bark, and dried. Modern research shows that the roots of goji berry is rich in a variety of terpenoids, sterols, phenols, phenylpropanoids, alkaloids, cyclic peptides, and other active ingredients (Chen et al., 2019; Jiang et al., 2020). Li et al., (2020) analyzed the quality of *Cortex Lycii* between different origins using kukoamines as the main evaluation index results showed that there were significant differences. The commercial specifications of Chinese herbal medicines classified the *Cortex Lycii* into sweet and salty *Cortex Lycii* depending on the salinity of the origin soil, and into three grades according to length and Wooden heart rate. It is also stated that *Cortex Lycii* has a poor skin and white heart, which is brittle and easy to break; while the counterfeit stem has a hard cortex and is more tough (China Association of Chinese Medicine, 2018). In recent years, apart from being used in a large number of compound formulations for the prevention and treatment of various diseases, the extract of *Cortex Lycii* is used to skin care products for whitening and anti‐aging (Zhang et al., 2019).

### 4.2 Goji berry leaves

The leaves of goji berry, known as the “Tian jing cao”, meaning sacred herb, which are rich in polysaccharides, phenolic compounds, alkaloids, and minerals. They have a variety of biological activities, such as antioxidant, anti‐inflammatory, anti‐diabetic, and anti‐microbial activities (Cui et al., 2020; Lee et al., 2021; Lei et al., 2022; Zhao et al., 2020). Nowadays, goji berry leaves have been widely consumed as tea and vegetables. Cultivate leaf kinds that can be utilized to produce wolfberry sprouts and sprouts tea industrially, such as those with enormous growth, strong adaptability, fresh and soft leaf buds, good flavor, and rich nutrition (Zhu et al., 2022). Korean researchers Ju et al. (2020) studied the effects of cultivation techniques and varieties on the quality of goji berry leaves. Among the 11 recommended varieties, “Mingan” showed the highest yield and rapid regeneration after cuttings. The amount of dry leaves obtained per 10 acres increased steadily from 106 kg on May 16 to 287 kg on June 20. Suitable cuttings date and cuttings length (about 60–70 cm) were important factors for its effective regeneration. Later cutting times were inappropriate due to the rigidity of the stems and the production of spines, which were difficult during plant harvest. The contents of betaine in goji berry leaves ranged from 1.43% to 2.63% and was significantly influenced by varieties and cuttings dates (Wang et al., 2020; Zhang, 2022). On the other hand, the phenolic compounds or flavonoids extracts of the leaves in goji berry has received much attention. Conidi et al. (2020) developed a water extraction combined with PES membrane technique for the separation of phenolic compounds from goji berry leaves. The results showed that the 1 kDa membrane showed the best performance in terms of polyphenol purification.

### 4.3 Goji berry branches

During the goji berry cultivation process, in order to achieve better aeration, light penetration, optimization of nutrients, reproductive growth, energy distribution, etc., pruning branches during spring and winter annually is a must. The pruning process produces a large amount of goji berry branches, of which only a very small part of the cuttings are used for seedling expansion, the rest are scattered as waste, or burned. The annual production of goji berry branch waste is more than 200,000 t in Ningxia only. Therefore, waste branches of goji berry have become one of the most important agricultural wastes generated in the process of goji berry cultivation. Research on the use of the branches is mainly focused on basalization, fodder, fertilization, and heat source (Li et al., 2021). Qu et al., (2019) used microorganisms to ferment the branches. It was found that goji berry branches can effectively accelerate the substrate process, shortening the fermentation time and improving the fermentation efficiency. Wang et al. (2019) fermented goji berry branches by mixing a lactic acid coccus and yeast, supplemented with nutrients such as bran, and finally made goji berry branch into biological feed.

### 4.4 Goji berry bee pollen

Goji berry bee pollen is another type of available by‐products. Our Group estimated that there is more than 120,000 t of potential production capacity of Chinese goji berry bee pollen in China each year. And the nutritional value of goji berry bee pollen is high and has good prospect of development (Yan et al., 2014). The contents of total soluble sugar, protein, total polyphenols, total flavonoids, and glucose in goji berry bee pollen are 44.8% w/w, 25.0% w/w, 22.95 mg/g, 21.17 mg/g, and 15.2% w/w, respectively (Zhou et al., 2018). The effect of goji berry bee pollen polysaccharides (WPPs) on tumors arising from xenografted DU145 prostate cancer cells was evaluated, and WPPs were found to induce apoptosis of DU145 cells in vitro and in vivo, while reducing the tumor weight and volume (Ran et al., 2020). The extraction of flavonoids from goji berry bee pollen was performed by ultrasonic‐assisted extraction, showing that the average value of total flavonoid contents obtained by the optimized extraction process was 3.03% w/w (Ran et al., 2012). In addition, goji berry honey is a unique honey variety in northwest China. He studied that goji berry honey has strong reductive ability, DPPH free radical scavenging activity, and antioxidant capacity (He, 2019).

### 4.5 Utilization of the residual fruit of goji berry

The residual fruit in goji berry also known as “you guo”, which is a local saying. Due to improper heat or air drying, improper storage, over‐mature, or harvesting in rain, the fresh fruit may be oxidized and becomes oily, in which the color becomes dark purple, giving the appearance not welcomed in marketing (Ji, 2018). However, the taste of “you guo” remains as being fresh. Although “you guo” is required to be removed as a residual product, it is still rich in a variety of nutrients, such as polysaccharides of 2.10%–5.77% w/w in the residual fruit (Ma et al., 2015). At the same time, the goji berry seeds in the residual fruit can be further made into goji berry seed oil to achieve the purpose of comprehensive utilization.

## 5 SUMMARY

The medicinal and edible herb *Lycium barbarum* L. is a valuable resource. The fruit, stems, leaves, and roots can all be consumed or used medicinally. The goji berry industry has been growing rapidly in Ningxia, Xinjiang, Qinghai, Gansu, and other Chinese provinces in response to people's demand for health. After complete processing of the extracted active ingredients, the utilization of goji berry by‐products such as fruit juice, wine, and inferior fruit, as well as the roots, stems, and leaves, has come into notice during the wolfberry planting process. As a result, the development trend for wolfberry processing and utilization is high‐efficiency, high‐value, and comprehensive utilization strategies. The deep utilization of wolfberry resources currently undergoes challenges due to issues like low comprehensive resource utilization, lack of high value‐added functional products, lack of multi‐stage co‐production processing and utilization technologies, and unstandardized production equipments. Future research and development may focus on deep‐processed products based on more functional substances, or further support the creation of standardized production technology, or to establish more efficient multi‐stage co‐production processing technology systems, or to expand the use of wolfberry by‐product resources. It is envisaged that new techniques described in this review will serve the thorough use of wolfberry resources and the superior growth of the wolfberry processing industry.

## AUTHOR CONTRIBUTIONS

**Jing Yu:** Validation (equal); writing – original draft (equal); writing – review and editing (equal). **Yamei Yan:** Project administration (equal); supervision (equal); visualization (equal). **Lutao Zhang:** Validation (equal); writing – review and editing (equal). **Jia Mi:** Investigation (equal). **Limei Yu:** Investigation (equal). **Fengfeng Zhang:** Project administration (equal); resources (equal); supervision (equal). **Lu Lu:** Investigation (equal). **Qing Luo:** Validation (equal). **Xiaoying Li:** Investigation (equal). **Xuan Zhou:** Validation (equal). **Youlong Cao:** Supervision (equal).

## CONFLICT OF INTEREST STATEMENT

The authors declare no competing interests.

## Data Availability

The data that support the findings of this study are openly available at https://doi.org/10.1002/fsn3.3677.
